# Transgender preventative health—chest/breast cancer screening

**DOI:** 10.3389/frhs.2024.1434536

**Published:** 2024-08-14

**Authors:** Valjean R. Bacot-Davis, Allison H. Moran

**Affiliations:** ^1^ Department of Medicine-Pediatrics, Stony Brook University Hospital, Stony Brook, NY, United States; ^2^ Department of Social Work, State University of New York at Albany, School of Social Welfare, Richardson Hall, Albany, NY, United States

**Keywords:** prevention, breast cancer, transgender (binary and non-binary), health prevention behavior, health practice approaches

## Abstract

Cancer mortality rates have decreased over the last 48 years attributable to standardized cancer screenings. These screenings were developed without deliberate inclusion of transgender and non-binary populations. While specialists are familiar regarding cancer screening in this distinct population, those in primary care might be more limited. As such, we aimed to develop a screening risk tool that combines the Breast Cancer Risk Assessment Tool (Gail model) with the updated American College of Radiology Appropriateness Criteria—Transgender Breast Cancer Screening, into an online questionnaire designed to accommodate primary care physicians performing routine health screenings to advise appropriate imaging and referral for this population. This new tool can be used for transgender chest/breast risk assessment whereas the Gail model alone was developed without transgender populations in mind, with the aim of early detection and cancer prevention in this historically underserved healthcare population.

## Introduction

Cancer mortality rates have decreased for all cancer types over the last 48 years since the passage of the National Cancer Act of 1971 as well as more widespread adoption of standardized cancer screening protocols (1, 2). However, these guidelines were developed based on large population studies without deliberate inclusion of the transgender and non-binary communities (2). Nonetheless, the decline in chest/breast, colorectal, and prostate cancers is partially attributable to increased detection and removal of premalignant and localized lesions prior to metastasis (2). While specialists in OB/GYN, urology, oncology, surgery, and radiology who regularly assume clinical care for members of the transgender and non-binary communities regularly review current guidelines and best practices regarding cancer screenings in these distinct populations, those in primary care might be more limited in familiarity due to their patient demographics, and less accustomed to the routine cancer screenings in relation to the transgender and non-binary population. There are some specific transgender cancer screening recommendations, but these are largely based on cisgender individuals and extrapolated as reasonable based on what organs systems remain *in situ* adjusted to transgender individuals (2).

Transgender healthcare inequalities represent a pressing and multifaceted issue at the intersection of healthcare, social justice, and human rights. Despite significant progress in recognizing and affirming the rights of transgender individuals, disparities in healthcare access and quality persist, often exacerbating existing societal inequities (3–7). From structural deficiencies within healthcare systems to provider bias and inadequate insurance coverage, these disparities undermine the health and well-being of transgender people, highlighting the urgent need for systemic reform and inclusive, culturally competent care (3, 8–10). The history of transgender health is complex and has evolved over time alongside societal attitudes, medical advancements, and advocacy efforts. The medicalization of transgender identity began in the early 20th century, with the development of sexology and endocrinology (11). Early medical interventions included hormone therapy and surgeries (12). Post-World War II: The mid-20th century saw increased medical interest in transgender health, but treatment options remained limited and often pathologized transgender identities (12–14). Many transgender individuals faced discrimination and stigma within medical settings (15) 1970s–1980s: The emergence of the modern LGBTQ + rights movement brought increased visibility to transgender issues. Activists began advocating for better healthcare access and standards of care for transgender individuals. However, medical gatekeeping and restrictive criteria for accessing transgender healthcare persisted (12). 1990s–2000s: The late 20th and early 21st centuries saw significant advancements in transgender healthcare. Standards of care, such as the Harry Benjamin Standards, were developed to guide medical professionals in providing transgender-affirming care. Surgical techniques improved, and hormone therapy became more accessible and refined. Legal recognition of transgender identities, including changes to gender markers on identification documents, became more common in many countries. However, disparities in healthcare access and discrimination persisted, particularly for transgender individuals from marginalized communities (15). The 21st century has seen continued progress in transgender healthcare, including increased insurance coverage for transgender-related care, greater cultural competency among healthcare providers, and advancements in surgical techniques. However, challenges remain, including barriers to accessing care, disparities in health outcomes, and ongoing stigma and discrimination (7, 11, 16, 17).

Chest/Breast cancer screening stands as a crucial cornerstone in the realm of health, serving as a proactive measure against one of the most prevalent and potentially devastating diseases (18). Its significance lies not only in early detection but also in the empowerment it offers individuals in taking charge of their health outcomes. By undergoing regular screening, individuals can detect abnormalities in chest/breast tissue at an early stage when treatment options are often more effective and less invasive (18, 19). Moreover, chest/breast cancer screening plays a pivotal role in raising awareness, promoting education, fostering a culture of preventive healthcare, and is a great accomplishment in public health. Nonetheless, chest/breast cancer screening for transgender individuals presents unique challenges and considerations (17, 20). Transgender individuals have unique medical and family histories as well as transition statuses, which can impact chest/breast cancer risk and screening recommendations. Transgender individuals can face barriers to healthcare, including discrimination and lack of access to gender-affirming care, which impact their ability to access breast cancer screening services. Healthcare providers should be aware of these barriers and work to create a supportive and inclusive environment for transgender patients.

Chest/breast cancer screening guidelines that healthcare providers currently use may not factor all the unique risk factors of transgender individuals. Overall, as seen in provider surveys performed by Azhir et al. (21) and Ufomata et al. (22), healthcare providers are generally unfamiliar with chest/breast cancer screening pertaining to transgender individuals. Furthermore, some providers do not feel comfortable providing care to the transgender community due to these highly individualized risk assessment needs. As such, we have worked to develop a questionnaire that makes it easier to assess individual risk amongst the transgender community based on the Gail Model and the American College or Radiology (ACR) Appropriateness Criteria, which will take providers minutes to use in order to know the most appropriate imagining modality to screen such patient for chest/breast cancer screening (18, 19, 23).

The Gail model was one of the earliest breast cancer risk assessment models, initially published in 1989. Its data was derived from 243,221 White women in the Breast Cancer Detection Demonstration Project between 1973 and 1980 in the United States and modified in 1992 by the National Surgical Adjuvant Breast and Bowel Project to estimate the absolute risk of developing only invasive breast cancer based on a combined proportional-hazards regression model and other risk factors. Since then, updates have been made for more accurate estimates for Black women, Asian and Pacific Islander women, and Hispanic women. This modified tool is available in the National Cancer Institute's (NCI) Breast Cancer Risk Assessment Tool at https://bcrisktool.cancer.gov (24). The Gail Model does not include specific risk assessments for transgender individuals or offer imaging guidelines based on the number of years on these individuals have been on hormone treatment or undergone applicable surgical interventions. The ACR Appropriateness Criteria for Transgender Breast Cancer Screening provides parallel recommendations based on the age of transgender individual, family history, personal risk factors, years of hormone treatment, and prior surgeries that are not opposed to the Gail Model, but have different categories based on age criteria that differ slightly from the Gail Model, sex designated at birth, gender identity, family and personal risk factors, years on hormone treatment, and prior surgeries.

It is important for healthcare providers to discuss chest/breast cancer screening with transgender patients in a sensitive and respectful manner. Some transgender individuals may feel uncomfortable or dysphoric about breast examinations or mammograms (25, 26). Providers should be mindful of these concerns and work with patients to develop a screening plan that feels both safe and comfortable. Although epidemiological information on the prevalence of breast cancer amongst transgender men and transgender women is limited, a 2023 systematic review estimates the risk of female-to-male transgender individuals as higher than cisgender men but lower than cisgender women [standardized incidence ratio (SIR) 63.4 vs. 0.42], while male-to-female transgender women are at higher risk compared to cisgender men and lower risk than cisgender women [SIR 22.5 vs. 0.30] (27). Both groups are at higher risk compared with cisgender men and lower risk than cisgender women. Conversely, it appears from this study that transgender men are at higher risk of developing chest/breast cancer than are transgender women when compared to cisgender women [SIR 0.42 vs. 0.30]. This study also acknowledges the lack of defined guidelines for this population (27). Overall, providing inclusive and culturally competent care is essential for ensuring that transgender individuals receive the most appropriate chest/breast cancer screening and support. This includes addressing barriers to healthcare access, respecting patient preferences, and utilizing screening guidelines as appropriate.

## Methods

### Transgender chest/breast cancer screening questionnaire

**Figure 1:**
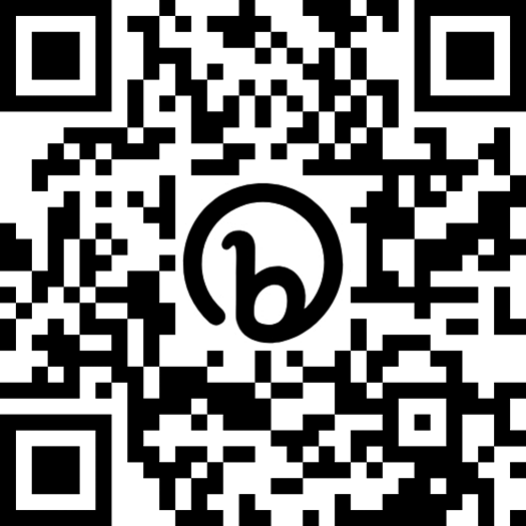
Transgender chest/breast cancer screening questionnaire. Link to the 16 yes-no screening questionnaire for transgender individuals with guideline screening recommendations based on responses: https://bit.ly/3p0EL6W.

The Practice Guidelines from the 2021 ACR Appropriateness Criteria—Transgender Breast Cancer Screening were used in conjunction with the Gail Model to create a 16 yes-no screening questionnaire. PHP (Hypertext Preprocessor) code was written based on the Yes/No input to create a pathway with echo response recommendations of imaging based on both ACR Appropriateness and Gail Model guidelines. Link to screening questionnaire: https://bit.ly/3p0EL6W. A QR Code to the transgender chest/breast cancer screening questionnaire is also provided in Figure 1.

### Chest/breast cancer screening table and educational video

A simplified table was also created and discussed at the end of an education video reviewing the best imaging modality to screen for breast/chest cancer amongst the transgender population (Table 1). This table was designed to fit on one page and can be easily displayed in primary care offices. A video review of the table was also recorded for quick review of current guidelines for screening this unique population. Link to educational video: https://bit.ly/4cKceGt (Supplementary Video S1).

**Table 1:** Simplified table reviewing the best imaging modality to screen for breast/chest cancer amongst the transgender population.

	Transwoman		Transman
Variant 1	Age: ≥40 Past or current hormone use ≥5 years Average risk (Gail model) Mammography may be appropriate	Variant 5	Age: any Double bilateral mastectomy Any risk (Gail model) Screening usually not appropriate
Variant 2	Age: ≥25 Past or current hormone use ≥5 years Higher than average risk (Gail model) Mammography usually appropriate	Variant 6	Age: ≥40 No surgery or breast reduction surgery Average risk (Gail model) Mammography usually appropriate
Variant 3	Age: any Past or current hormone use <5 years Average risk (Gail model) Screening usually not appropriate	Variant 7	Age: ≥30 No surgery or breast reduction surgery Intermediate risk (Gail model) Mammography usually appropriate Ultrasound may be appropriate
Variant 4	Age: ≥25 Past or current hormone use <5 years Higher than average risk (Gail model) Mammography may be appropriate	Variant 8	Age: ≥25 No surgery or breast reduction surgery High risk (Gail model) Mammography usually appropriate Ultrasound may be appropriate

## Results

Hypertext Preprocessor (PHP) code (Supplementary File S1) was used to develop a website (Figure 1) and underwent perception testing from members of the transgender community to verify correct modality responses based on age, risk factors, and family history with the same input into the Gail Model and ACR Appropriateness Criteria individually. To assess perceived ease of use as follows: Strongly Agree, Moderately Agree, Somewhat Agree, Neutral, Moderately Disagree, Strongly Disagree. N 60, Mean 3.29, St. deviation 0.69, with 70% choosing Strongly Agree to Somewhat Agree. Lastly, a more simplified one-page table of current ACR transgender chest/breast cancer screening guidelines and video were created for easy review (Table 1).

## Discussion

The Gail Model is a statistical tool used to estimate cisgender women's risk of developing breast cancer based on age, age at menarche, age at first live birth, number of breast biopsies, history of atypical hyperplasia, and number of first-degree relatives with breast cancer. While the model was initially developed based on the data of White women from the National Institutes of Health's Surveillance, Epidemiology, and End Results (SEER) Program data, the model has since been revised and is now validated for estimating risk in White cisgender women, Black/African American cisgender women, Hispanic cisgender women, and for Asian and Pacific Islander cisgender women in the United State and developed countries (18, 19, 28, 29). However, a limitation is that the tool likely underestimates risk for Black/African American cisgender women with previous biopsies, Hispanic women born outside the United States, and has limited and possibly inaccurate data on American Indian/Alaska Native women due to limited participants. Another limitation of the Gail Model is that it does not include all potential risk factors, such as genetic mutations (e.g., BRCA1 and BRCA2), detailed family history beyond first-degree relatives, or lifestyle factors like diet, exercise, and alcohol consumption. It is most accurate predicting short-term risk (5 years) rather than long-term risk, and is designed for assessing initial breast cancer risk, not the risk of recurrence. Nonetheless, statistically validated studies have shown that while both transgender women and transgender men have higher risks of breast cancer compared to cisgender men, both groups collectively have a lower risk than do cisgender women (27). Additionally, the Gail model may over-estimate their breast cancer risk. As such, large-scale studies are needed into the development of a breast cancer risk assessment tool for the transgender population. A lack of existing large transgender randomized prospective trial designs, small sample sizes, recruitment bias, short study duration, and high subject dropout rates are all possible barrier to development of such a risk assessment.

The ACR Appropriateness Criteria for transgender breast cancer extrapolates known evidence-based biological factors to help determine risk. Large cisgender studies have shown that exogenous hormones, in particular estrogen and progestin, increase breast cancer risk in cisgender, postmenopausal women. In cisgender men, high estrogen levels are also a recognized risk factors for developing breast cancer. Still, some experts disagree with the ACR appropriateness recommendation against breast cancer screening for transgender men who have undergone “top surgery” or double bilateral mastectomy. Furthermore, existing literature is limited by inconsistent doses and lengths of exposure to exogenous hormones, small sample sizes, and short follow-ups in this population. In the absence large clinical data, transgender health experts and professional societies established guidelines as recommendations, such as the Endocrine Society recommending screening transgender women with the same frequency as cisgender women beginning at age 40 (30). Unfortunately, while existing recommendations are based on extrapolations from large cisgender studies or limited retrospective studies of transgender individuals, there are increasing signifiers of assigned/designated gender at birth and current gender identity that may incorporate larger cohorts of transgender individuals in national and longitudinal cancer risk repositories for future analysis. The combined ACR Transgender Appropriateness Criteria and Gail Model chest/breast risk stratification inherently contains all the limitations and strengths of the Gail model and current professional expert society recommendations based on the limited cohort and longitudinal studies we currently have for the transgender population.

Strengths of the Gail Model is that it is evidence-based and validated. It was developed based on large, well-established epidemiological studies. The updated Gail Model now more accurately assesses breast cancer risk amongst racial/ethnic minority groups after more accurate risk estimates for Black/African American women using data from the Contraceptive and Reproductive Experiences Study and SEER study; Asian and Pacific Islander women in the US using data from the Asian American Breast Cancer Study and SEER study; and Hispanic women using data from the San Francisco Bay Area Breast Cancer Study, the California Cancer Registry, the California SEER Program, and SEER study (31, 32). The Gail model is also relatively simple to use and is accessible online to both healthcare providers and patients to discuss their risk (28). Strengths of the ACR Appropriateness Criteria for transgender individuals include acknowledgment that the healthcare needs of transgender patients might differ significantly from cisgender individuals, considers additional individual risk factors such as hormone duration, surgical history, family history, offers inclusivity in medical imaging to assess risk of chest/breast cancer, and reflects a commitment to health equity by addressing the specific needs of historically underserved transgender individuals. Recommendations for future research would be to analyze racial minorities amongst the transgender population to assess their overall risk of chest/breast cancer, like done for the Gail Model after its initial data analysis and risk stratifications were developed for White women.

Systematic review has shown that both transgender men and transgender women are at lower risk of chest/breast cancer when compared to cisgender women (27). As such, the Gail Model in conjunction with ACR expert opinion can be presumed to be at least, if not more sensitive, although less specific, due to lower incidence in the transgender population compared with cisgender women. The RAND/UCLA Appropriateness Method and Grading of Recommendations Assessment, Development, and Evaluation or GRADE have been used to rate the appropriateness of imaging and treatment procedures for specific transgender clinical scenarios. In these instances, due to small population sizes and lack of longitudinal study times, expert opinion can be used to supplement current available evidence for imaging recommendations and treatment (23). In the lack of longitudinal large studies of transgender individual and their development of chest/breast cancer, and even less data on transgender racial/ethnic minorities, GRADE appropriateness expert opinion is validated to use in these instances. Therefore, as this new model is built based on the validated expert opinion ACR Transgender Appropriateness Criteria and statistically validated Gail Model, GRADE appropriateness for the use of the combined model is reasonable until such longitudinal data is available and analyzed for this minority group.

The adjusted Gail Model has a sensitivity of 0.709 and a specificity of 0.622 (33). Based on incidence and prevalence of chest/breast cancer in the transgender population, the current model is projected to have a lower estimated sensitivity and higher estimated specificity, Female-to-Male (FtM) (SIR = 0.42) and Male-to-Female (MtF) (SIR = 0.30), compared to cisgender women. Due to lack of long-term longitudinal studies in the transgender population, the current ACR Appropriateness Criteria expert opinion and Gail Model are appropriate. Special considerations in this transgender and gender non-conforming/gender diverse population encompasses unexpected emotional discomfort with screening that might preclude initial or longitudinal follow-up. A previous survey found that ultrasound examinations (49%) and mammography (33%) caused high rates of emotional discomfort in transgender and gender non-binary patients undergoing imaging studies, while less invasive imaging studies such as MRI were associated with less emotional distress (24%) (34). This survey suggests that this population might be more likely to undergo and follow-up with MRI screening, if clinically indicated for chest/breast cancer screening. This survey also found that many transgender and gender non-binary respondents found the radiology environment to be unwelcoming (45%), and noticed the lack of affirming lesbian, gay, bisexual, transgender, and queer representation in magazines, posters, and educational materials in the environment. Such inclusion might also assist with longitudinal follow-up.

Another recent study found that combining the Gail model with the Breast Imaging Reporting and Data System (BI-RADS) for predicting the malignancy of breast nodules showed better diagnostic efficiency than either the BI-RADS or Gail model alone [Area Under Curve (AUC) 0.98 vs. 0.80, *p* < 0.001; AUC 0.98 vs. 0.55, *p* < 0.001] and demonstrated a higher specificity than the BI-RADS [91.3% vs. 59.4%, *p* < 0.001] (35). Combining the Gail model with the ACR Appropriateness Criteria for transgender breast cancer could arguably also demonstrate a higher specificity than the ACR Appropriateness Criteria for transgender breast cancer alone. Risk profiles would be derived via a questionnaire in a national or international Transgender Health Study bi-annual survey, and the incident of cancer cases in participants compared with cisgender controls, to determine the 5- and 10-year sensitivity and specificity of the combined model. The data from hundreds of cases and controls would need to be gathered. Compared to the known incidence and prevalence of chest/breast cancer amongst the cisgender and transgender population, the model would likely have a lower estimated sensitivity and higher estimated specificity, although racial/ethnic disparities in detection for the transgender population would need to be further studied.

Potential ethical considerations including data privacy and informed consent must be considered when gathering patient and participant healthcare information. The data input for the combined ACR Transgender Appropriateness Criteria and Gail Model chest/breast risk stratification is not stored in any repository. The development PHP code is available and demonstrates the lack of data collection, lack of tracking of computer IP address, lack of cookies, and lack of collection of other identifying information. Furthermore, data repositories that collect and provide de-identified annual healthcare information for the LGBTQ + community are available through The Pride Study (36), which has already partnered with researchers to make such information available in an ethical way.

The LGBTQ + community is diverse and encompasses a vast array of gender identities and sexual orientations. This community also includes people of all ethnicities/racial categories and socioeconomic backgrounds. This intersectionality impacts both access and delivery of health care. Barriers are multifactorial and include stigma, discrimination, insurance coverage, lack of health care providers training, and lack of research. Transgender individuals often face barriers in healthcare, including lack of tailored guidelines and discrimination, which can result in suboptimal chest/breast cancer screening and care. Inclusive and culturally competent healthcare practices are essential for improving outcomes in this population. Due to small sample sizes, study results involving transgender disease incidence must often be interpreted with caution due to heterogeneity in screening participation compounded by a need to disaggregate data to account for intersecting identities of racial/ethnic minorities (37). Many national health surveys need revision to use gender-inclusive language or allow patients to separately self-report their sex designated/assigned at birth and current gender identity, hormone use, and gender-affirming chest/breast surgery that are concordant with gender identity. Accessing health care remains a challenge for gender-diverse individuals because many health care systems adhere to a gender binary model.

Here, we developed the first combined ACR Transgender Appropriateness Criteria and Gail Model chest/breast risk stratification into one new tool developed with the goal to improving chest/breast cancer early detection and early treatment in this population. The ACR Appropriateness imaging guidelines are specified by age, hormone, and surgical history with lifetime risk stratification according to the verified Gail model. Importantly, in some studies, mass mammography screening can improve early cancer detection by as much as 15%–35% (38). Risk-based screening reduces healthcare cost while maintaining intensive screening for the highest-risk women. The cost-effectiveness of breast cancer screening in the CDC National Breast and Cervical Cancer Early Detection Program was cost-effective among the target population of low-income, uninsured women aged 40–64 years. The base-case incremental cost-effectiveness ratios were $51,754/quality-adjusted life-years compared to no program and $50,223/quality-adjusted life-years compared to no screening (39).

Overall, transgender chest/breast cancer screening requires an approach that considers each person's medical history, gender identity, surgical history, family history, and unique healthcare needs. By promoting inclusivity, providing education, and engaging in respectful communication, healthcare providers can ensure that transgender individuals receive the care and support they need to maintain their health. To our knowledge, this is the first time that a screening questionnaire has been developed combining the Gail Model with the 2021 ACR Appropriateness Criteria for Transgender Chest/Breast cancer screening for healthcare providers to use to order/recommended the correct imaging modality for chest/breast cancer screening. It is our hope that this questionnaire tool in addition to the simplified one-page table will be used to increase provider comfort at routine primary care cancer screening and image ordering, as well as reduce mortality amongst the transgender population.

## Data Availability

The original contributions presented in the study are included in the article/Supplementary Material, further inquiries can be directed to the corresponding author.

## Supplementary material

Supplementary File 1 PHP code used to develop the questionnaire and responses for the Transgender Chest/Breast Cancer Screening Questionnaire. Link to PHP code: https://github.com/vbacotdavis/Transgender-PHP

Supplementary Video 1 Short video reviewing current guidelines and recommendations for screening transgender individuals for chest/breast cancer (Video 1). Of note, video case vignettes are fabricated for educational purposes and do not relate to real patients.
