# Supplementary material for: Transgender preventative health—chest/breast cancer screening
Source: Front Health Serv. 2024 Aug 14;4:1434536. doi: 10.3389/frhs.2024.1434536 (PMC11349637; doi:10.3389/frhs.2024.1434536)
Supplement: Supplementary File 1 — PHP code used to develop the questionnaire and responses for the Transgender Chest/Breast Cancer Screening Questionnaire. Link to PHP code: https://github.com/vbacotdavis/Transgender-PHP [file Table1.docx]

<form action="/submit-form" method="POST">

  <p>Question 1: Transfeminine?</p>

  <label><input type="radio" name="q1" value="yes">Yes</label>

  <label><input type="radio" name="q1" value="no">No</label>

  <p>Question 2: Transmasculine?</p>

  <label><input type="radio" name="q2" value="yes">Yes</label>

  <label><input type="radio" name="q2" value="no">No</label>

  <p>Question 3: &lt; 25 years old?</p>

  <label><input type="radio" name="q3" value="yes">Yes</label>

  <label><input type="radio" name="q3" value="no">No</label>

  <p>Question 4: 25-29 years old?</p>

  <label><input type="radio" name="q4" value="yes">Yes</label>

  <label><input type="radio" name="q4" value="no">No</label>

  <p>Question 5: 30-39 years old?</p>

  <label><input type="radio" name="q5" value="yes">Yes</label>

  <label><input type="radio" name="q5" value="no">No</label>

  <p>Question 6: &#8805; 40 years old?</p>

  <label><input type="radio" name="q6" value="yes">Yes</label>

  <label><input type="radio" name="q6" value="no">No</label>

  <p>Question 7: &lt; 5 years total exogenous estrogen exposure</p>

  <label><input type="radio" name="q7" value="yes">Yes</label>

  <label><input type="radio" name="q7" value="no">No</label>

  <p>Question 8: &#8805; 5 years total exogenous estrogen exposure</p>

  <label><input type="radio" name="q8" value="yes">Yes</label>

  <label><input type="radio" name="q8" value="no">No</label>

  <p>Question 9: Bilateral Mastectomy?</p>

  <label><input type="radio" name="q9" value="yes">Yes</label>

  <label><input type="radio" name="q9" value="no">No</label>

  <p>Question 10: Yes if no chest surgery or Yes if only a reduction mammoplasty</p>

  <label><input type="radio" name="q10" value="yes">Yes</label>

  <label><input type="radio" name="q10" value="no">No</label>

  <p>Question 11: Any personal history of chest/breast cancer or chest irradiation at 10-30 years of age?</p>

  <label><input type="radio" name="q11" value="yes">Yes</label>

  <label><input type="radio" name="q11" value="no">No</label>

  <p>Question 12: Any family history of chest/breast cancer or family genetic pre-disposition to chest/breast cancer?</p>

  <label><input type="radio" name="q12" value="yes">Yes</label>

  <label><input type="radio" name="q12" value="no">No</label>

  <p>Question 13: &lt; 15% lifetime risk of chest/breast cancer? (ex. Gail model)</p>

  <label><input type="radio" name="q13" value="yes">Yes</label>

  <label><input type="radio" name="q13" value="no">No</label>

  <p>Question 14: 15-20% lifetime risk of chest/breast cancer? (ex. Gail model)</p>

  <label><input type="radio" name="q14" value="yes">Yes</label>

  <label><input type="radio" name="q14" value="no">No</label>

  <p>Question 15: &gt; 20% lifetime risk of chest/breast cancer? (ex. Gail model)</p>

  <label><input type="radio" name="q15" value="yes">Yes</label>

  <label><input type="radio" name="q15" value="no">No</label>

  <p>Question 16: Genetically untested with a first-degree relative with genetic predisposition to chest/breast cancer?</p>

  <label><input type="radio" name="q16" value="yes">Yes</label>

  <label><input type="radio" name="q16" value="no">No</label>

  <button type="submit">Submit</button>

</form>

<p>Please complete Gail Model Risk score prior to submission.</p>

<p>

<Body>

 <div class="a">Screening recommendations based on: Expert Panel on Breast Imaging, Brown A, Lourenco AP, et al. ACR Appropriateness Criteria® Transgender Breast Cancer Screening. *J Am Coll Radiol*. 2021;18(11S):S502-S515. doi:10.1016/j.jacr.2021.09.005 </div> <br>

</Body>

</p>

<?php

if ($_SERVER['REQUEST_METHOD'] == 'POST') {

  $q1 = $_POST['q1'];

  $q2 = $_POST['q2'];

  $q3 = $_POST['q3'];

  $q4 = $_POST['q4'];

  $q5 = $_POST['q5'];

  $q6 = $_POST['q6'];

  $q7 = $_POST['q7'];

  $q8 = $_POST['q8'];

  $q9 = $_POST['q9'];

  $q10 = $_POST['q10'];

  $q11 = $_POST['q11'];

  $q12 = $_POST['q12'];

  $q13 = $_POST['q13'];

  $q14 = $_POST['q14'];

  $q15 = $_POST['q15'];

  $q16 = $_POST['q16'];

   if($q1 == 'yes' && $q2 == 'no' && $q3 == 'no' && $q4 == 'no' && $q5 == 'no' && $q6 == 'yes' && $q7 == 'no' && $q8 == 'yes' && $q9 == 'no' && $q10 == 'yes' && $q11 == 'no' && $q12 == 'no' && $q13 == 'yes' && $q14 == 'no' && $q15 == 'no' && $q16 == 'no') {

   echo '<p>Response: Digital breast tomosynthesis or Mammography: screening MAY BE appropriate. US breast and MRI breast w/wo contrast: screening USUALLY NOT appropriate</p>';

} else if($q1 == 'yes' && $q2 == 'no' && $q3 == 'no' && $q4 == 'yes' && $q5 == 'yes' && $q6 == 'yes' && $q7 == 'no' && $q8 == 'yes' && $q9 == 'no' && $q10 == 'yes' && $q11 == 'yes' && $q12 == 'yes' && $q13 == 'no' && $q14 == 'no' && $q15 == 'no' && $q16 == 'yes') {

   echo '<p>Response: Digital breast tomosynthesis or Mammography: screening USUALLY appropriate. US breast and MRI breast w/wo contrast: screening USUALLY NOT appropriate</p>';

} else if($q1 == 'yes' && $q2 == 'no' && $q3 == 'no' && $q4 == 'yes' && $q5 == 'no' && $q6 == 'no' && $q7 == 'no' && $q8 == 'yes' && $q9 == 'no' && $q10 == 'yes' && $q11 == 'yes' && $q12 == 'no' && $q13 == 'no' && $q14 == 'no' && $q15 == 'no' && $q16 == 'no') {

   echo '<p>Response: Digital breast tomosynthesis or Mammography: screening USUALLY appropriate. US breast and MRI breast w/wo contrast: screening USUALLY NOT appropriate</p>';

} else if($q1 == 'yes' && $q2 == 'no' && $q3 == 'no' && $q4 == 'yes' && $q5 == 'no' && $q6 == 'no' && $q7 == 'no' && $q8 == 'yes' && $q9 == 'no' && $q10 == 'yes' && $q11 == 'no' && $q12 == 'yes' && $q13 == 'no' && $q14 == 'no' && $q15 == 'no' && $q16 == 'no') {

   echo '<p>Response: Digital breast tomosynthesis or Mammography: screening USUALLY appropriate. US breast and MRI breast w/wo contrast: screening USUALLY NOT appropriate</p>';

} else if($q1 == 'yes' && $q2 == 'no' && $q3 == 'no' && $q4 == 'yes' && $q5 == 'no' && $q6 == 'no' && $q7 == 'no' && $q8 == 'yes' && $q9 == 'no' && $q10 == 'yes' && $q11 == 'no' && $q12 == 'no' && $q13 == 'no' && $q14 == 'yes' && $q15 == 'no' && $q16 == 'no') {

   echo '<p>Response: Digital breast tomosynthesis or Mammography: screening USUALLY appropriate. US breast and MRI breast w/wo contrast: screening USUALLY NOT appropriate</p>';

} else if($q1 == 'yes' && $q2 == 'no' && $q3 == 'no' && $q4 == 'yes' && $q5 == 'no' && $q6 == 'no' && $q7 == 'no' && $q8 == 'yes' && $q9 == 'no' && $q10 == 'yes' && $q11 == 'no' && $q12 == 'no' && $q13 == 'no' && $q14 == 'no' && $q15 == 'yes' && $q16 == 'no') {

   echo '<p>Response: Digital breast tomosynthesis or Mammography: screening USUALLY appropriate. US breast and MRI breast w/wo contrast: screening USUALLY NOT appropriate</p>';

} else if($q1 == 'yes' && $q2 == 'no' && $q3 == 'no' && $q4 == 'yes' && $q5 == 'no' && $q6 == 'no' && $q7 == 'no' && $q8 == 'yes' && $q9 == 'no' && $q10 == 'yes' && $q11 == 'no' && $q12 == 'no' && $q13 == 'no' && $q14 == 'no' && $q15 == 'no' && $q16 == 'yes') {

   echo '<p>Response: Digital breast tomosynthesis or Mammography: screening USUALLY appropriate. US breast and MRI breast w/wo contrast: screening USUALLY NOT appropriate</p>';

} else if($q1 == 'yes' && $q2 == 'no' && $q3 == 'no' && $q4 == 'yes' && $q5 == 'no' && $q6 == 'no' && $q7 == 'no' && $q8 == 'yes' && $q9 == 'no' && $q10 == 'yes' && $q11 == 'no' && $q12 == 'no' && $q13 == 'no' && $q14 == 'yes' && $q15 == 'no' && $q16 == 'no') {

   echo '<p>Response: Digital breast tomosynthesis or Mammography: screening USUALLY appropriate. US breast and MRI breast w/wo contrast: screening USUALLY NOT appropriate</p>';

} else if($q1 == 'yes' && $q2 == 'no' && $q3 == 'no' && $q4 == 'no' && $q5 == 'yes' && $q6 == 'yes' && $q7 == 'no' && $q8 == 'yes' && $q9 == 'no' && $q10 == 'yes' && $q11 == 'yes' && $q12 == 'yes' && $q13 == 'no' && $q14 == 'no' && $q15 == 'no' && $q16 == 'yes') {

   echo '<p>Response: Digital breast tomosynthesis or Mammography: screening USUALLY appropriate. US breast and MRI breast w/wo contrast: screening USUALLY NOT appropriate</p>';

} else if($q1 == 'yes' && $q2 == 'no' && $q3 == 'no' && $q4 == 'no' && $q5 == 'yes' && $q6 == 'no' && $q7 == 'no' && $q8 == 'yes' && $q9 == 'no' && $q10 == 'yes' && $q11 == 'yes' && $q12 == 'no' && $q13 == 'no' && $q14 == 'no' && $q15 == 'no' && $q16 == 'no') {

   echo '<p>Response: Digital breast tomosynthesis or Mammography: screening USUALLY appropriate. US breast and MRI breast w/wo contrast: screening USUALLY NOT appropriate</p>';

} else if($q1 == 'yes' && $q2 == 'no' && $q3 == 'no' && $q4 == 'no' && $q5 == 'yes' && $q6 == 'no' && $q7 == 'no' && $q8 == 'yes' && $q9 == 'no' && $q10 == 'yes' && $q11 == 'no' && $q12 == 'yes' && $q13 == 'no' && $q14 == 'no' && $q15 == 'no' && $q16 == 'no') {

   echo '<p>Response: Digital breast tomosynthesis or Mammography: screening USUALLY appropriate. US breast and MRI breast w/wo contrast: screening USUALLY NOT appropriate</p>';

} else if($q1 == 'yes' && $q2 == 'no' && $q3 == 'no' && $q4 == 'no' && $q5 == 'yes' && $q6 == 'no' && $q7 == 'no' && $q8 == 'yes' && $q9 == 'no' && $q10 == 'yes' && $q11 == 'no' && $q12 == 'no' && $q13 == 'no' && $q14 == 'yes' && $q15 == 'no' && $q16 == 'no') {

   echo '<p>Response: Digital breast tomosynthesis or Mammography: screening USUALLY appropriate. US breast and MRI breast w/wo contrast: screening USUALLY NOT appropriate</p>';

} else if($q1 == 'yes' && $q2 == 'no' && $q3 == 'no' && $q4 == 'no' && $q5 == 'yes' && $q6 == 'no' && $q7 == 'no' && $q8 == 'yes' && $q9 == 'no' && $q10 == 'yes' && $q11 == 'no' && $q12 == 'no' && $q13 == 'no' && $q14 == 'no' && $q15 == 'yes' && $q16 == 'no') {

   echo '<p>Response: Digital breast tomosynthesis or Mammography: screening USUALLY appropriate. US breast and MRI breast w/wo contrast: screening USUALLY NOT appropriate</p>';

} else if($q1 == 'yes' && $q2 == 'no' && $q3 == 'no' && $q4 == 'no' && $q5 == 'yes' && $q6 == 'no' && $q7 == 'no' && $q8 == 'yes' && $q9 == 'no' && $q10 == 'yes' && $q11 == 'no' && $q12 == 'no' && $q13 == 'no' && $q14 == 'no' && $q15 == 'no' && $q16 == 'yes') {

   echo '<p>Response: Digital breast tomosynthesis or Mammography: screening USUALLY appropriate. US breast and MRI breast w/wo contrast: screening USUALLY NOT appropriate</p>';

} else if($q1 == 'yes' && $q2 == 'no' && $q3 == 'no' && $q4 == 'no' && $q5 == 'yes' && $q6 == 'no' && $q7 == 'no' && $q8 == 'yes' && $q9 == 'no' && $q10 == 'yes' && $q11 == 'no' && $q12 == 'no' && $q13 == 'no' && $q14 == 'yes' && $q15 == 'no' && $q16 == 'no') {

   echo '<p>Response: Digital breast tomosynthesis or Mammography: screening USUALLY appropriate. US breast and MRI breast w/wo contrast: screening USUALLY NOT appropriate</p>';

} else if($q1 == 'yes' && $q2 == 'no' && $q3 == 'no' && $q4 == 'yes' && $q5 == 'yes' && $q6 == 'no' && $q7 == 'no' && $q8 == 'yes' && $q9 == 'no' && $q10 == 'yes' && $q11 == 'yes' && $q12 == 'yes' && $q13 == 'no' && $q14 == 'no' && $q15 == 'no' && $q16 == 'yes') {

   echo '<p>Response: Digital breast tomosynthesis or Mammography: screening USUALLY appropriate. US breast and MRI breast w/wo contrast: screening USUALLY NOT appropriate</p>';

} else if($q1 == 'yes' && $q2 == 'no' && $q3 == 'no' && $q4 == 'no' && $q5 == 'no' && $q6 == 'yes' && $q7 == 'no' && $q8 == 'yes' && $q9 == 'no' && $q10 == 'yes' && $q11 == 'yes' && $q12 == 'no' && $q13 == 'no' && $q14 == 'no' && $q15 == 'no' && $q16 == 'no') {

   echo '<p>Response: Digital breast tomosynthesis or Mammography: screening USUALLY appropriate. US breast and MRI breast w/wo contrast: screening USUALLY NOT appropriate</p>';

} else if($q1 == 'yes' && $q2 == 'no' && $q3 == 'no' && $q4 == 'no' && $q5 == 'no' && $q6 == 'yes' && $q7 == 'no' && $q8 == 'yes' && $q9 == 'no' && $q10 == 'yes' && $q11 == 'no' && $q12 == 'yes' && $q13 == 'no' && $q14 == 'no' && $q15 == 'no' && $q16 == 'no') {

   echo '<p>Response: Digital breast tomosynthesis or Mammography: screening USUALLY appropriate. US breast and MRI breast w/wo contrast: screening USUALLY NOT appropriate</p>';

} else if($q1 == 'yes' && $q2 == 'no' && $q3 == 'no' && $q4 == 'no' && $q5 == 'no' && $q6 == 'yes' && $q7 == 'no' && $q8 == 'yes' && $q9 == 'no' && $q10 == 'yes' && $q11 == 'no' && $q12 == 'no' && $q13 == 'no' && $q14 == 'yes' && $q15 == 'no' && $q16 == 'no') {

   echo '<p>Response: Digital breast tomosynthesis or Mammography: screening USUALLY appropriate. US breast and MRI breast w/wo contrast: screening USUALLY NOT appropriate</p>';

} else if($q1 == 'yes' && $q2 == 'no' && $q3 == 'no' && $q4 == 'no' && $q5 == 'no' && $q6 == 'yes' && $q7 == 'no' && $q8 == 'yes' && $q9 == 'no' && $q10 == 'yes' && $q11 == 'no' && $q12 == 'no' && $q13 == 'no' && $q14 == 'no' && $q15 == 'yes' && $q16 == 'no') {

   echo '<p>Response: Digital breast tomosynthesis or Mammography: screening USUALLY appropriate. US breast and MRI breast w/wo contrast: screening USUALLY NOT appropriate</p>';

} else if($q1 == 'yes' && $q2 == 'no' && $q3 == 'no' && $q4 == 'no' && $q5 == 'no' && $q6 == 'yes' && $q7 == 'no' && $q8 == 'yes' && $q9 == 'no' && $q10 == 'yes' && $q11 == 'no' && $q12 == 'no' && $q13 == 'no' && $q14 == 'no' && $q15 == 'no' && $q16 == 'yes') {

   echo '<p>Response: Digital breast tomosynthesis or Mammography: screening USUALLY appropriate. US breast and MRI breast w/wo contrast: screening USUALLY NOT appropriate</p>';

} else if($q1 == 'yes' && $q2 == 'no' && $q3 == 'no' && $q4 == 'no' && $q5 == 'no' && $q6 == 'yes' && $q7 == 'no' && $q8 == 'yes' && $q9 == 'no' && $q10 == 'yes' && $q11 == 'no' && $q12 == 'no' && $q13 == 'no' && $q14 == 'yes' && $q15 == 'no' && $q16 == 'no') {

   echo '<p>Response: Digital breast tomosynthesis or Mammography: screening USUALLY appropriate. US breast and MRI breast w/wo contrast: screening USUALLY NOT appropriate</p>';

} else if($q1 == 'yes' && $q2 == 'no' && ($q3 == 'yes' || $q3 == 'no') && ($q4 == 'yes' || $q4 == 'no') && ($q5 == 'yes' || $q5 == 'no') && ($q6 == 'yes' || $q6 == 'no') && $q7 == 'yes' && $q8 == 'no' && ($q9 == 'yes' || $q9 == 'no') && ($q10 == 'yes' || $q10 == 'no') && $q11 == 'no' && $q12 == 'no' && $q13 == 'yes' && $q14 == 'no' && $q15 == 'no' && $q16 == 'no') {

   echo '<p>Response: Chest/Breast cancer screening USUALLY NOT appropriate</p>';

} else if($q1 == 'yes' && $q2 == 'no' && $q3 == 'no' && $q4 == 'yes' && $q5 == 'yes' && $q6 == 'yes' && $q7 == 'yes' && $q8 == 'no' && $q9 == 'no' && $q10 == 'yes' && $q11 == 'yes' && $q12 == 'yes' && $q13 == 'no' && $q14 == 'no' && $q15 == 'no' && $q16 == 'yes') {

   echo '<p>Response: Digital breast tomosynthesis or Mammography: screening MAY BE appropriate. US breast and MRI breast w/wo contrast: screening USUALLY NOT appropriate</p>';

} else if($q1 == 'yes' && $q2 == 'no' && $q3 == 'no' && $q4 == 'yes' && $q5 == 'no' && $q6 == 'no' && $q7 == 'yes' && $q8 == 'no' && $q9 == 'no' && $q10 == 'yes' && $q11 == 'yes' && $q12 == 'no' && $q13 == 'no' && $q14 == 'no' && $q15 == 'no' && $q16 == 'no') {

   echo '<p>Response: Digital breast tomosynthesis or Mammography: screening MAY BE appropriate. US breast and MRI breast w/wo contrast: screening USUALLY NOT appropriate</p>';

} else if($q1 == 'yes' && $q2 == 'no' && $q3 == 'no' && $q4 == 'yes' && $q5 == 'no' && $q6 == 'no' && $q7 == 'yes' && $q8 == 'no' && $q9 == 'no' && $q10 == 'yes' && $q11 == 'no' && $q12 == 'yes' && $q13 == 'no' && $q14 == 'no' && $q15 == 'no' && $q16 == 'no') {

   echo '<p>Response: Digital breast tomosynthesis or Mammography: screening MAY BE appropriate. US breast and MRI breast w/wo contrast: screening USUALLY NOT appropriate</p>';

} else if($q1 == 'yes' && $q2 == 'no' && $q3 == 'no' && $q4 == 'yes' && $q5 == 'no' && $q6 == 'no' && $q7 == 'yes' && $q8 == 'no' && $q9 == 'no' && $q10 == 'yes' && $q11 == 'no' && $q12 == 'no' && $q13 == 'no' && $q14 == 'yes' && $q15 == 'no' && $q16 == 'no') {

   echo '<p>Response: Digital breast tomosynthesis or Mammography: screening MAY BE appropriate. US breast and MRI breast w/wo contrast: screening USUALLY NOT appropriate</p>';

} else if($q1 == 'yes' && $q2 == 'no' && $q3 == 'no' && $q4 == 'yes' && $q5 == 'no' && $q6 == 'no' && $q7 == 'yes' && $q8 == 'no' && $q9 == 'no' && $q10 == 'yes' && $q11 == 'no' && $q12 == 'no' && $q13 == 'no' && $q14 == 'no' && $q15 == 'yes' && $q16 == 'no') {

   echo '<p>Response: Digital breast tomosynthesis or Mammography: screening MAY BE appropriate. US breast and MRI breast w/wo contrast: screening USUALLY NOT appropriate</p>';

} else if($q1 == 'yes' && $q2== 'no' && $q3 == 'no' && $q4 == 'yes' && $q5 == 'no' && $q6 == 'no' && $q7 == 'yes' && $q8 == 'no' && $q9 == 'no' && $q10 == 'yes' && $q11 == 'no' && $q12 == 'no' && $q13 == 'no' && $q14 == 'no' && $q15 == 'no' && $q16 == 'yes') {

   echo '<p>Response: Digital breast tomosynthesis or Mammography: screening MAY BE appropriate. US breast and MRI breast w/wo contrast: screening USUALLY NOT appropriate</p>';

} else if($q1 == 'yes' && $q2 == 'no' && $q3 == 'no' && $q4 == 'yes' && $q5 == 'no' && $q6 == 'no' && $q7 == 'yes' && $q8 == 'no' && $q9 == 'no' && $q10 == 'yes' && $q11 == 'no' && $q12 == 'no' && $q13 == 'no' && $q14 == 'yes' && $q15 == 'no' && $q16 == 'no') {

   echo '<p>Response: Digital breast tomosynthesis or Mammography: screening MAY BE appropriate. US breast and MRI breast w/wo contrast: screening USUALLY NOT appropriate</p>';

} else if($q1 == 'yes' && $q2 == 'no' && $q3 == 'no' && $q4 == 'no' && $q5 == 'yes' && $q6 == 'yes' && $q7 == 'yes' && $q8 == 'no' && $q9 == 'no' && $q10 == 'yes' && $q11 == 'yes' && $q12 == 'yes' && $q13 == 'no' && $q14 == 'no' && $q15 == 'no' && $q16 == 'yes') {

   echo '<p>Response: Digital breast tomosynthesis or Mammography: screening MAY BE appropriate. US breast and MRI breast w/wo contrast: screening USUALLY NOT appropriate</p>';

} else if($q1 == 'yes' && $q2 == 'no' && $q3 == 'no' && $q4 == 'no' && $q5 == 'yes' && $q6 == 'no' && $q7 == 'yes' && $q8 == 'no' && $q9 == 'no' && $q10 == 'yes' && $q11 == 'yes' && $q12 == 'no' && $q13 == 'no' && $q14 == 'no' && $q15 == 'no' && $q16 == 'no') {

   echo '<p>Response: Digital breast tomosynthesis or Mammography: screening MAY BE appropriate. US breast and MRI breast w/wo contrast: screening USUALLY NOT appropriate</p>';

} else if($q1 == 'yes' && $q2 == 'no' && $q3 == 'no' && $q4 == 'no' && $q5 == 'yes' && $q6 == 'no' && $q7 == 'yes' && $q8 == 'no' && $q9 == 'no' && $q10 == 'yes' && $q11 == 'no' && $q12 == 'yes' && $q13 == 'no' && $q14 == 'no' && $q15 == 'no' && $q16 == 'no') {

   echo '<p>Response: Digital breast tomosynthesis or Mammography: screening MAY BE appropriate. US breast and MRI breast w/wo contrast: screening USUALLY NOT appropriate</p>';

} else if($q1 == 'yes' && $q2 == 'no' && $q3 == 'no' && $q4 == 'no' && $q5 == 'yes' && $q6 == 'no' && $q7 == 'yes' && $q8 == 'no' && $q9 == 'no' && $q10 == 'yes' && $q11 == 'no' && $q12 == 'no' && $q13 == 'no' && $q14 == 'yes' && $q15 == 'no' && $q16 == 'no') {

   echo '<p>Response: Digital breast tomosynthesis or Mammography: screening MAY BE appropriate. US breast and MRI breast w/wo contrast: screening USUALLY NOT appropriate</p>';

} else if($q1 == 'yes' && $q2 == 'no' && $q3 == 'no' && $q4 == 'no' && $q5 == 'yes' && $q6 == 'no' && $q7 == 'yes' && $q8 == 'no' && $q9 == 'no' && $q10 == 'yes' && $q11 == 'no' && $q12 == 'no' && $q13 == 'no' && $q14 == 'no' && $q15 == 'yes' && $q16 == 'no') {

   echo '<p>Response: Digital breast tomosynthesis or Mammography: screening MAY BE appropriate. US breast and MRI breast w/wo contrast: screening USUALLY NOT appropriate</p>';

} else if($q1 == 'yes' && $q2 == 'no' && $q3 == 'no' && $q4 == 'no' && $q5 == 'yes' && $q6 == 'no' && $q7 == 'yes' && $q8 == 'no' && $q9 == 'no' && $q10 == 'yes' && $q11 == 'no' && $q12 == 'no' && $q13 == 'no' && $q14 == 'no' && $q15 == 'no' && $q16 == 'yes') {

   echo '<p>Response: Digital breast tomosynthesis or Mammography: screening MAY BE appropriate. US breast and MRI breast w/wo contrast: screening USUALLY NOT appropriate</p>';

} else if($q1 == 'yes' && $q2 == 'no' && $q3 == 'no' && $q4 == 'no' && $q5 == 'yes' && $q6 == 'no' && $q7 == 'yes' && $q8 == 'no' && $q9 == 'no' && $q10 == 'yes' && $q11 == 'no' && $q12 == 'no' && $q13 == 'no' && $q14 == 'yes' && $q15 == 'no' && $q16 == 'no') {

   echo '<p>Response: Digital breast tomosynthesis or Mammography: screening MAY BE appropriate. US breast and MRI breast w/wo contrast: screening USUALLY NOT appropriate</p>';

} else if($q1 == 'yes' && $q2 == 'no' && $q3 == 'no' && $q4 == 'yes' && $q5 == 'yes' && $q6 == 'no' && $q7 == 'yes' && $q8 == 'no' && $q9 == 'no' && $q10 == 'yes' && $q11 == 'yes' && $q12 == 'yes' && $q13 == 'no' && $q14 == 'no' && $q15 == 'no' && $q16 == 'yes') {

   echo '<p>Response: Digital breast tomosynthesis or Mammography: screening MAY BE appropriate. US breast and MRI breast w/wo contrast: screening USUALLY NOT appropriate</p>';

} else if($q1 == 'yes' && $q2 == 'no' && $q3 == 'no' && $q4 == 'no' && $q5 == 'no' && $q6 == 'yes' && $q7 == 'yes' && $q8 == 'no' && $q9 == 'no' && $q10 == 'yes' && $q11 == 'yes' && $q12 == 'no' && $q13 == 'no' && $q14 == 'no' && $q15 == 'no' && $q16 == 'no') {

   echo '<p>Response: Digital breast tomosynthesis or Mammography: screening MAY BE appropriate. US breast and MRI breast w/wo contrast: screening USUALLY NOT appropriate</p>';

} else if($q1 == 'yes' && $q2 == 'no' && $q3 == 'no' && $q4 == 'no' && $q5 == 'no' && $q6 == 'yes' && $q7 == 'yes' && $q8 == 'no' && $q9 == 'no' && $q10 == 'yes' && $q11 == 'no' && $q12 == 'yes' && $q13 == 'no' && $q14 == 'no' && $q15 == 'no' && $q16 == 'no') {

   echo '<p>Response: Digital breast tomosynthesis or Mammography: screening MAY BE appropriate. US breast and MRI breast w/wo contrast: screening USUALLY NOT appropriate</p>';

} else if($q1 == 'yes' && $q2 == 'no' && $q3 == 'no' && $q4 == 'no' && $q5 == 'no' && $q6 == 'yes' && $q7 == 'yes' && $q8 == 'no' && $q9 == 'no' && $q10 == 'yes' && $q11 == 'no' && $q12 == 'no' && $q13 == 'no' && $q14 == 'yes' && $q15 == 'no' && $q16 == 'no') {

   echo '<p>Response: Digital breast tomosynthesis or Mammography: screening MAY BE appropriate. US breast and MRI breast w/wo contrast: screening USUALLY NOT appropriate</p>';

} else if($q1 == 'yes' && $q2 == 'no' && $q3 == 'no' && $q4 == 'no' && $q5 == 'no' && $q6 == 'yes' && $q7 == 'yes' && $q8 == 'no' && $q9 == 'no' && $q10 == 'yes' && $q11 == 'no' && $q12 == 'no' && $q13 == 'no' && $q14 == 'no' && $q15 == 'yes' && $q16 == 'no') {

   echo '<p>Response: Digital breast tomosynthesis or Mammography: screening MAY BE appropriate. US breast and MRI breast w/wo contrast: screening USUALLY NOT appropriate</p>';

} else if($q1 == 'yes' && $q2 == 'no' && $q3 == 'no' && $q4 == 'no' && $q5 == 'no' && $q6 == 'yes' && $q7 == 'yes' && $q8 == 'no' && $q9 == 'no' && $q10 == 'yes' && $q11 == 'no' && $q12 == 'no' && $q13 == 'no' && $q14 == 'no' && $q15 == 'no' && $q16 == 'yes') {

   echo '<p>Response: Digital breast tomosynthesis or Mammography: screening MAY BE appropriate. US breast and MRI breast w/wo contrast: screening USUALLY NOT appropriate</p>';

} else if($q1 == 'yes' && $q2 == 'no' && $q3 == 'no' && $q4 == 'no' && $q5 == 'no' && $q6 == 'yes' && $q7 == 'yes' && $q8 == 'no' && $q9 == 'no' && $q10 == 'yes' && $q11 == 'no' && $q12 == 'no' && $q13 == 'no' && $q14 == 'yes' && $q15 == 'no' && $q16 == 'no') {

   echo '<p>Response: Digital breast tomosynthesis or Mammography: screening MAY BE appropriate. US breast and MRI breast w/wo contrast: screening USUALLY NOT appropriate</p>';

} else if($q1 == 'no' && $q2 == 'yes' && ($q3 == 'yes' || $q3 == 'no') && ($q4 == 'yes' || $q4 == 'no') && ($q5 == 'yes' || $q5 == 'no') && ($q6 == 'yes' || $q6 == 'no') && $q7 == 'yes' && $q8 == 'no' && $q9 == 'yes' && $q10 == 'no' && ($q11 == 'yes' || $q11 == 'no') && ($q12 == 'yes' || $q12 == 'no') && ($q13 == 'yes' || $q13 == 'no') && ($q14 == 'yes' || $q14 == 'no') && ($q15 == 'yes' || $q15 == 'no') && ($q16 == 'yes' || $q16 == 'no')) {

   echo '<p>Response: Chest/Breast cancer screening USUALLY NOT appropriate</p>';

} else if($q1 == 'no' && $q2 == 'no' && ($q3 == 'yes' || $q3 == 'no') && ($q4 == 'yes' || $q4 == 'no') && ($q5 == 'yes' || $q5 == 'no') && ($q6 == 'yes' || $q6 == 'no') && ($q7 == 'yes' || $q7 == 'no') && ($q8 == 'yes' || $q8 == 'no') && ($q9 == 'yes' || $q9 == 'no') && ($q10 == 'yes' || $q10 == 'no') && ($q11 == 'yes' || $q11 == 'no') && ($q12 == 'yes' || $q12 == 'no') && ($q13 == 'yes' || $q13 == 'no') && ($q14 == 'yes' || $q14 == 'no') && ($q15 == 'yes' || $q15 == 'no') && ($q16 == 'yes' || $q16 == 'no')) {

   echo '<p>Response: Weight risk factors based on age, personal history, family history, exogenous estrogen use total or greater than 5 years, menstrual history (if applicable), birth history (if applicable), and surgical history</p>';

} else if($q1 == 'no' && $q2 == 'yes' && $q3 == 'no' && $q4 == 'no' && $q5 == 'no' && $q6 == 'yes' && ($q7 == 'yes' || $q7 == 'no') && ($q8 == 'yes' || $q8 == 'no') && $q9 == 'no' && $q10 == 'yes' && $q11 == 'no' && $q12 == 'no' && $q13 == 'yes' && $q14 == 'no' && $q15 == 'no' && $q16 == 'no') {

   echo '<p>Response: Digital breast tomosynthesis or Mammography: screening USUALLY appropriate. US breast and MRI breast w/wo contrast: screening USUALLY NOT appropriate</p>';

} else if($q1 == 'no' && $q2 == 'yes' && $q3 == 'no' && $q4 == 'no' && $q5 == 'yes' && $q6 == 'no' && ($q7 == 'yes' || $q7 == 'no') && ($q8 == 'yes' || $q8 == 'no') && $q9 == 'no' && $q10 == 'yes' && $q11 == 'yes' && $q12 == 'no' && $q13 == 'no' && $q14 == 'no' && $q15 == 'no' && $q16 == 'no') {

   echo '<p>Response: Digital breast tomosynthesis or Mammography: screening USUALLY appropriate. US breast or MRI Breast w and wo IV contrast: screening MAY BE appropriate. MRI breast without IV contrast: screening USUALLY NOT appropriate</p>';

} else if($q1 == 'no' && $q2 == 'yes' && $q3 == 'no' && $q4 == 'no' && $q5 == 'yes' && $q6 == 'no' && ($q7 == 'yes' || $q7 == 'no') && ($q8 == 'yes' || $q8 == 'no') && $q9 == 'no' && $q10 == 'yes' && $q11 == 'no' && $q12 == 'yes' && $q13 == 'no' && $q14 == 'no' && $q15 == 'no' && $q16 == 'no') {

   echo '<p>Response: Digital breast tomosynthesis or Mammography: screening USUALLY appropriate. US breast or MRI Breast w and wo IV contrast: screening MAY BE appropriate. MRI breast without IV contrast: screening USUALLY NOT appropriate</p>';

} else if($q1 == 'no' && $q2 == 'yes' && $q3 == 'no' && $q4 == 'no' && $q5 == 'yes' && $q6 == 'no' && ($q7 == 'yes' || $q7 == 'no') && ($q8 == 'yes' || $q8 == 'no') && $q9 == 'no' && $q10 == 'yes' && $q11 == 'no' && $q12 == 'no' && $q13 == 'no' && $q14 == 'yes' && $q15 == 'no' && $q16 == 'no') {

   echo '<p>Response: Digital breast tomosynthesis or Mammography: screening USUALLY appropriate. US breast or MRI Breast w and wo IV contrast: screening MAY BE appropriate. MRI breast without IV contrast: screening USUALLY NOT appropriate</p>';

} else if($q1 == 'no' && $q2 == 'yes' && $q3 == 'no' && $q4 == 'no' && $q5 == 'yes' && $q6 == 'no' && ($q7 == 'yes' || $q7 == 'no') && ($q8 == 'yes' || $q8 == 'no') && $q9 == 'no' && $q10 == 'yes' && $q11 == 'no' && $q12 == 'no' && $q13 == 'no' && $q14 == 'no' && $q15 == 'no' && $q16 == 'no') {

   echo '<p>Response: Digital breast tomosynthesis or Mammography: screening USUALLY appropriate. US breast or MRI Breast w and wo IV contrast: screening MAY BE appropriate. MRI breast without IV contrast: screening USUALLY NOT appropriate</p>';

} else if($q1 == 'no' && $q2 == 'yes' && $q3 == 'no' && $q4 == 'no' && $q5 == 'yes' && $q6 == 'no' && ($q7 == 'yes' || $q7 == 'no') && ($q8 == 'yes' || $q8 == 'no') && $q9 == 'no' && $q10 == 'yes' && $q11 == 'no' && $q12 == 'no' && $q13 == 'no' && $q14 == 'no' && $q15 == 'no' && $q16 == 'yes') {

   echo '<p>Response: Digital breast tomosynthesis or Mammography: screening USUALLY appropriate. US breast or MRI Breast w and wo IV contrast: screening MAY BE appropriate. MRI breast without IV contrast: screening USUALLY NOT appropriate</p>';

} else if($q1 == 'no' && $q2 == 'yes' && $q3 == 'no' && $q4 == 'no' && ($q5 == 'yes' || $q5 == 'no') && ($q6 == 'yes' || $q6 == 'no') && ($q7 == 'yes' || $q7 == 'no') && ($q8 == 'yes' || $q8 == 'no') && $q9 == 'no' && $q10 == 'yes' && $q11 == 'yes' && $q12 == 'no' && $q13 == 'no' && $q14 == 'no' && $q15 == 'no' && $q16 == 'no') {

   echo '<p>Response: Digital breast tomosynthesis or Mammography: screening USUALLY appropriate. US breast or MRI Breast w and wo IV contrast: screening MAY BE appropriate. MRI breast without IV contrast: screening USUALLY NOT appropriate</p>';

} else if($q1 == 'no' && $q2 == 'yes' && $q3 == 'no' && $q4 == 'no' && ($q5 == 'yes' || $q5 == 'no') && ($q6 == 'yes' || $q6 == 'no') && ($q7 == 'yes' || $q7 == 'no') && $q8 == 'yes||no' && $q9 == 'no' && $q10 == 'yes' && $q11 == 'yes' && $q12 == 'yes' && $q13 == 'no' && $q14 == 'no' && $q15 == 'no' && $q16 == 'no') {

   echo '<p>Response: Digital breast tomosynthesis or Mammography: screening USUALLY appropriate. US breast or MRI Breast w and wo IV contrast: screening MAY BE appropriate. MRI breast without IV contrast: screening USUALLY NOT appropriate</p>';

} else if($q1 == 'no' && $q2 == 'yes' && $q3 == 'no' && $q4 == 'no' && ($q5 == 'yes' || $q5 == 'no') && ($q6 == 'yes' || $q6 == 'no') && ($q7 == 'yes' || $q7 == 'no') && ($q8 == 'yes' || $q8 == 'no') && $q9 == 'no' && $q10 == 'yes' && $q11 == 'yes' && $q12 == 'yes' && $q13 == 'no' && $q14 == 'yes' && $q15 == 'no' && $q16 == 'no') {

   echo '<p>Response: Digital breast tomosynthesis or Mammography: screening USUALLY appropriate. US breast or MRI Breast w and wo IV contrast: screening MAY BE appropriate. MRI breast without IV contrast: screening USUALLY NOT appropriate</p>';

} else if($q1 == 'no' && $q2 == 'yes' && $q3 == 'no' && $q4 == 'no' && ($q5 == 'yes' || $q5 == 'no') && ($q6 == 'yes' || $q6 == 'no') && ($q7 == 'yes' || $q7 == 'no') && ($q8 == 'yes' || $q8 == 'no') && $q9 == 'no' && $q10 == 'yes' && $q11 == 'yes' && $q12 == 'no' && $q13 == 'no' && $q14 == 'no' && $q15 == 'no' && $q16 == 'no') {

   echo '<p>Response: Digital breast tomosynthesis or Mammography: screening USUALLY appropriate. US breast or MRI Breast w and wo IV contrast: screening MAY BE appropriate. MRI breast without IV contrast: screening USUALLY NOT appropriate</p>';

} else if($q1 == 'no' && $q2 == 'yes' && $q3 == 'no' && $q4 == 'no' && $q5 == 'yes||no' && $q6 == 'yes||no' && $q7 == 'yes||no' && $q8 == 'yes||no' && $q9 == 'no' && $q10 == 'yes' && $q11 == 'yes' && $q12 == 'no' && $q13 == 'no' && $q14 == 'no' && $q15 == 'no' && $q16 == 'yes') {

   echo '<p>Response: Digital breast tomosynthesis or Mammography: screening USUALLY appropriate. US breast or MRI Breast w and wo IV contrast: screening MAY BE appropriate. MRI breast without IV contrast: screening USUALLY NOT appropriate</p>';

} else if($q1 == 'no' && $q2 == 'yes' && $q3 == 'no' && $q4 == 'no' && ($q5 == 'yes' || $q5 == 'no') && ($q6 == 'yes' || $q6 == 'no') && ($q7 == 'yes' || $q7 == 'no') && ($q8 == 'yes' || $q8 == 'no') && $q9 == 'no' && $q10 == 'yes' && $q11 == 'yes' && $q12 == 'no' && $q13 == 'no' && $q14 == 'yes' && $q15 == 'no' && $q16 == 'yes') {

   echo '<p>Response: Digital breast tomosynthesis or Mammography: screening USUALLY appropriate. US breast or MRI Breast w and wo IV contrast: screening MAY BE appropriate. MRI breast without IV contrast: screening USUALLY NOT appropriate</p>';

} else if($q1 == 'no' && $q2 == 'yes' && $q3 == 'no' && $q4 == 'no' && ($q5 == 'yes' || $q5 == 'no') && ($q6 == 'yes' || $q6 == 'no') && ($q7 == 'yes' || $q7 == 'no') && ($q8 == 'yes' || $q8 == 'no') && $q9 == 'no' && $q10 == 'yes' && $q11 == 'yes' && $q12 == 'no' && $q13 == 'no' && $q14 == 'no' && $q15 == 'no' && $q16 == 'yes') {

   echo '<p>Response: Digital breast tomosynthesis or Mammography: screening USUALLY appropriate. US breast or MRI Breast w and wo IV contrast: screening MAY BE appropriate. MRI breast without IV contrast: screening USUALLY NOT appropriate</p>';

} else if($q1 == 'no' && $q2 == 'yes' && $q3 == 'no' && $q4 == 'no' && ($q5 == 'yes' || $q5 == 'no') && ($q6 == 'yes' || $q6 == 'no') && ($q7 == 'yes' || $q7 == 'no') && ($q8 == 'yes' || $q8 == 'no') && $q9 == 'no' && $q10 == 'yes' && $q11 == 'no' && $q12 == 'yes' && $q13 == 'no' && $q14 == 'yes' && $q15 == 'no' && $q16 == 'yes') {

   echo '<p>Response: Digital breast tomosynthesis or Mammography: screening USUALLY appropriate. US breast or MRI Breast w and wo IV contrast: screening MAY BE appropriate. MRI breast without IV contrast: screening USUALLY NOT appropriate</p>';

   } else if($q1 == 'no' && $q2 == 'yes' && $q3 == 'no' && $q4 == 'no' && ($q5 == 'yes' || $q5 == 'no') && ($q6 == 'yes' || $q6 == 'no') && ($q7 == 'yes' || $q7 == 'no') && ($q8 == 'yes' || $q8 == 'no') && $q9 == 'no' && $q10 == 'yes' && $q11 == 'no' && $q12 == 'yes' && $q13 == 'no' && $q14 == 'no' && $q15 == 'no' && $q16 == 'yes') {

   echo '<p>Response: Digital breast tomosynthesis or Mammography: screening USUALLY appropriate. US breast or MRI Breast w and wo IV contrast: screening MAY BE appropriate. MRI breast without IV contrast: screening USUALLY NOT appropriate</p>';

} else if($q1 == 'no' && $q2 == 'yes' && $q3 == 'no' && $q4 == 'no' && ($q5 == 'yes' || $q5 == 'no') && ($q6 == 'yes' || $q6 == 'no') && ($q7 == 'yes' || $q7 == 'no') && ($q8 == 'yes' || $q8 == 'no') && $q9 == 'no' && $q10 == 'yes' && $q11 == 'no' && $q12 == 'no' && $q13 == 'no' && $q14 == 'no' && $q15 == 'no' && $q16 == 'yes') {

   echo '<p>Response: Digital breast tomosynthesis or Mammography: screening USUALLY appropriate. US breast or MRI Breast w and wo IV contrast: screening MAY BE appropriate. MRI breast without IV contrast: screening USUALLY NOT appropriate</p>';

} else if($q1 == 'no' && $q2 == 'yes' && $q3 == 'no' && ($q4 == 'yes' || $q4 == 'no') && ($q5 == 'yes' || $q5 == 'no') && ($q6 == 'yes' || $q6 == 'no') && ($q7 == 'yes' || $q7 == 'no') && ($q8 == 'yes' || $q8 == 'no') && $q9 == 'no' && $q10 == 'yes' && $q11 == 'yes' && $q12 == 'no' && $q13 == 'no' && $q14 == 'no' && $q15 == 'no' && $q16 == 'no') {

   echo '<p>Response: Digital breast tomosynthesis or Mammography or MRI breast w and wo IV contrast: screening USUALLY appropriate. US breast: screening MAY BE appropriate. MRI breast without IV contrast: screening USUALLY NOT appropriate</p>';

} else if($q1 == 'no' && $q2 == 'yes' && $q3 == 'no' && ($q4 == 'yes' || $q4 == 'no') && ($q5 == 'yes' || $q5 == 'no') && ($q6 == 'yes' || $q6 == 'no') && ($q7 == 'yes' || $q7 == 'no') && ($q8 == 'yes' || $q8 == 'no') && $q9 == 'no' && $q10 == 'yes' && $q11 == 'no' && $q12 == 'yes' && $q13 == 'no' && $q14 == 'no' && $q15 == 'yes' && $q16 == 'no') {

   echo '<p>Response: Digital breast tomosynthesis or Mammography or MRI breast w and wo IV contrast: screening USUALLY appropriate. US breast: screening MAY BE appropriate. MRI breast without IV contrast: screening USUALLY NOT appropriate</p>';

} else if($q1 == 'no' && $q2 == 'yes' && $q3 == 'no' && ($q4 == 'yes' || $q4 == 'no') && ($q5 == 'yes' || $q5 == 'no') && ($q6 == 'yes' || $q6 == 'no') && ($q7 == 'yes' || $q7 == 'no') && ($q8 == 'yes' || $q8 == 'no') && $q9 == 'no' && $q10 == 'yes' && $q11 == 'no' && $q12 == 'no' && $q13 == 'no' && $q14 == 'no' && $q15 == 'yes' && $q16 == 'no') {

   echo '<p>Response: Digital breast tomosynthesis or Mammography or MRI breast w and wo IV contrast: screening USUALLY appropriate. US breast: screening MAY BE appropriate. MRI breast without IV contrast: screening USUALLY NOT appropriate</p>';

} else if($q1 == 'no' && $q2 == 'yes' && $q3 == 'no' && ($q4 == 'yes' || $q4 == 'no') && ($q5 == 'yes' || $q5 == 'no') && ($q6 == 'yes' || $q6 == 'no') && ($q7 == 'yes' || $q7 == 'no') && ($q8 == 'yes' || $q8 == 'no') && $q9 == 'no' && $q10 == 'yes' && $q11 == 'no' && $q12 == 'no' && $q13 == 'no' && $q14 == 'no' && $q15 == 'no' && $q16 == 'yes') {

   echo '<p>Response: Digital breast tomosynthesis or Mammography or MRI breast w and wo IV contrast: screening USUALLY appropriate. US breast: screening MAY BE appropriate. MRI breast without IV contrast: screening USUALLY NOT appropriate</p>';

} else if($q1 == 'no' && $q2 == 'yes' && $q3 == 'no' && ($q4 == 'yes' || $q4 == 'no') && ($q5 == 'yes' || $q5 == 'no')  && ($q6 == 'yes' || $q6 == 'no') && ($q7 == 'yes' || $q7 == 'no') && ($q8 == 'yes' || $q8 == 'no') && $q9 == 'no' && $q10 == 'yes' && $q11 == 'yes' && $q12 == 'no' && $q13 == 'no' && $q14 == 'no' && $q15 == 'yes' && $q16 == 'no') {

   echo '<p>Response: Digital breast tomosynthesis or Mammography or MRI breast w and wo IV contrast: screening USUALLY appropriate. US breast: screening MAY BE appropriate. MRI breast without IV contrast: screening USUALLY NOT appropriate</p>';

   } else if($q1 == 'no' && $q2 == 'yes' && $q3 == 'no' && ($q4 == 'yes' || $q4 == 'no') && ($q5 == 'yes' || $q5 == 'no') && ($q6 == 'yes' || $q6 == 'no') && ($q7 == 'yes' || $q7 == 'no') && ($q8 == 'yes' || $q8 == 'no') && $q9 == 'no' && $q10 == 'yes' && $q11 == 'no' && $q12 == 'yes' && $q13 == 'no' && $q14 == 'no' && $q15 == 'no' && $q16 == 'yes') {

   echo '<p>Response: Digital breast tomosynthesis or Mammography or MRI breast w and wo IV contrast: screening USUALLY appropriate. US breast: screening MAY BE appropriate. MRI breast without IV contrast: screening USUALLY NOT appropriate!</p>';

} else if($q1 == 'no' && $q2 == 'yes' && $q3 == 'no' && ($q4 == 'yes' || $q4 == 'no') && ($q5 == 'yes' || $q5 == 'no') && ($q6 == 'yes' || $q6 == 'no') && ($q7 == 'yes' || $q7 == 'no') && ($q8 == 'yes' || $q8 == 'no') && $q9 == 'no' && $q10 == 'yes' && $q11 == 'no' && $q12 == 'yes' && $q13 == 'no' && $q14 == 'no' && $q15 == 'yes' && $q16 == 'no') {

   echo '<p>Response: Digital breast tomosynthesis or Mammography or MRI breast w and wo IV contrast: screening USUALLY appropriate. US breast: screening MAY BE appropriate. MRI breast without IV contrast: screening USUALLY NOT appropriate!</p>';

} else {

   echo '<p>Response: Invalid answers!</p>';

}

echo '<p>Thank you for your submission! Verify with ACR appropriateness criteria as needed.</p>';

  }

?>
